# Liquid Droplets as Emerging Biomaterials

**DOI:** 10.1021/accountsmr.3c00098

**Published:** 2023-07-13

**Authors:** Armando Huang, Lu Su

**Affiliations:** Leiden Academic Centre for Drug Research (LACDR), Leiden University, Einsteinweg 55, Leiden, 2333 CC The Netherlands

Liquid droplets, formed through noncovalent interactions of small molecules or macromolecules, have revolutionized our understanding of cellular organization and function since the discovery of biomolecular condensates in the cellular realm.^1^ These liquid droplets are generated via liquid–liquid phase separation (LLPS), referring to the spontaneous separation of (macro)molecules in distinct concentrated and diluted liquid phases driven by weak noncovalent interactions, such as electrostatic, π–π, cation−π, and hydrophobic interactions (Figure 1A). The reversible nature of these interactions makes the droplets dynamic, highly tunable, and responsive to environmental stimuli. Inspired by the natural process, this *Viewpoint* explores the potential of synthetic and reconstituted liquid droplets as novel biomaterials in drug delivery, diagnostics, immunomodulation, and particularly, model systems for condensate-modifying therapeutics (Figure 2).

**Figure 1:**
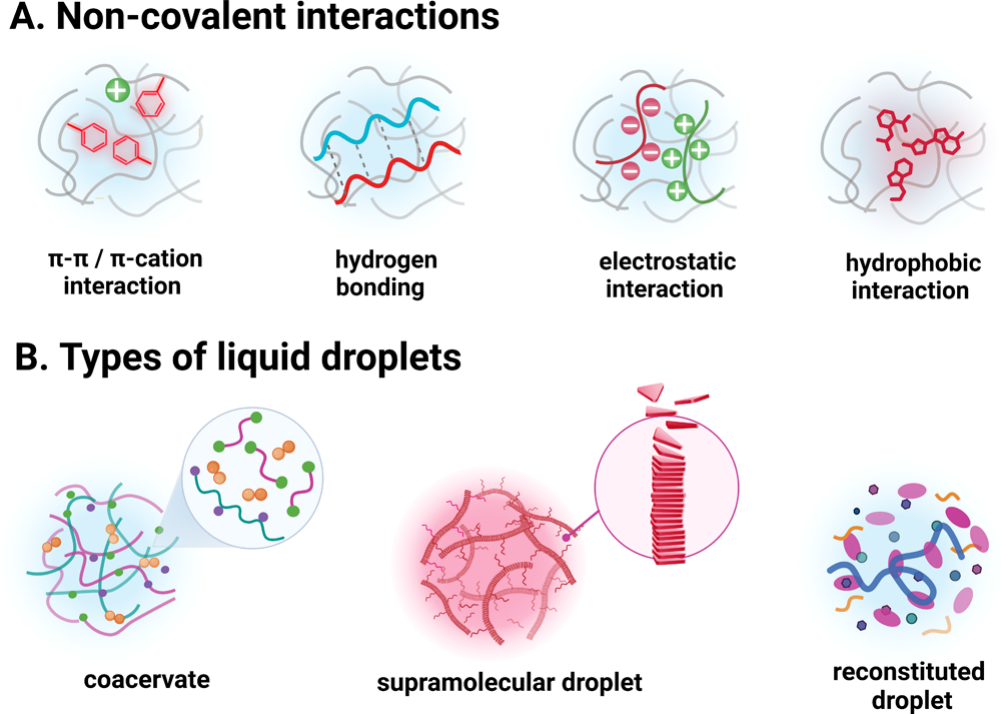
Schematic presentation of different (A) noncovalent interactions and (B) liquid droplets discussed in this *Viewpoint*. The figure was created with Biorender.com.

**Figure 2:**
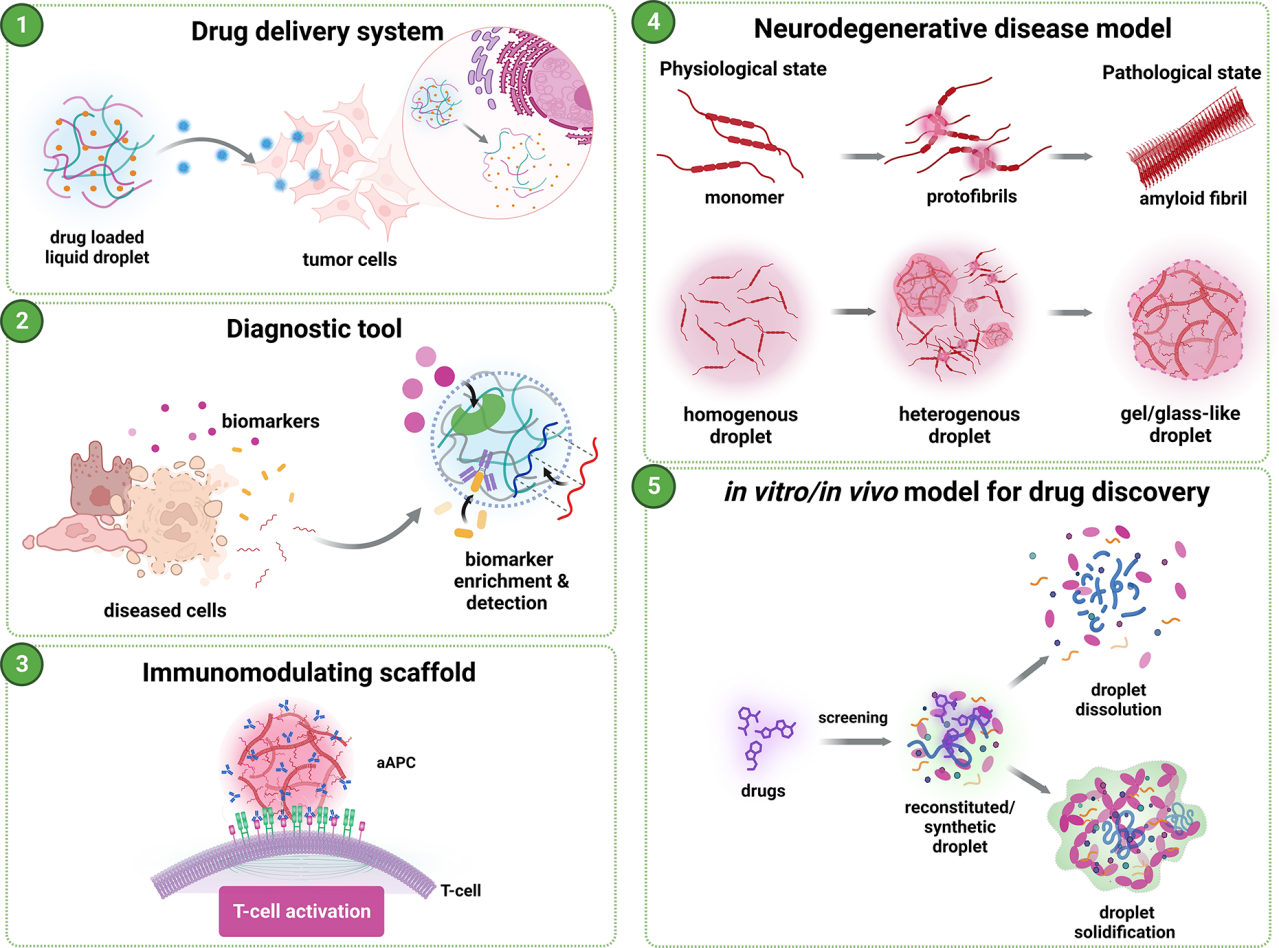
Potential applications of liquid droplets, such as (1) drug delivery systems, (2) diagnostic tools, (3) immunomodulating scaffolds, (4) neurodegenerative disease model, and (5) *in vitro* model for drug discovery. The figure is created with Biorender.com.

The liquid droplets discussed in this Viewpoint include (1) coacervates formed by synthetic polymers, such as the oppositely charged polypeptides, (2) supramolecular droplets generated by the interplay of the LLPS and supramolecular polymerization, and (3) reconstituted biomolecular condensates that consist of specific scaffold proteins and/or nucleic acids (Figure 1B).

## Drug Delivery System

Liquid droplets can dynamically recruit a wide range of therapeutics, such as small drugs, nucleic acids, proteins, and nanoparticles, driven by enhanced noncovalent interactions harbored inside, making them versatile delivery systems. Moreover, the dynamic nature enables triggered release/disassembly upon external cues like pH, salts, and enzymes. This property allows for targeted drug release to the body component of interest, thereby reducing off-target effects and enhancing the therapeutic efficacy. An example shows redox-responsive droplets could bypass the classical endocytic pathways, directly enter the cytoplasm, and undergo enzyme-triggered release, although the mechanism is not yet fully unraveled.^2^

To harness the full potential of these liquid droplets as drug delivery systems, crucial insights into the cellular uptake mechanisms are needed. Additionally, precise control over droplet properties, such as size, stability, therapeutics loading capacity, and *in vivo* release kinetics, needs to be achieved. In this regard, peptide-based droplets are particularly attractive owing to their biocompatibility and well-tailorable properties. Readers are highly recommended to read a recent *Comment* on this topic.^3^

## Diagnostics

Liquid droplets are gaining research attention in the field of diagnostics as innovative tools for biomarker enrichment and detection. The biosensing capability of liquid droplets encompasses the selective encapsulation and the subsequent enrichment of biomarkers (proteins, metabolites, nucleic acid, and extracellular vesicles) within the droplet through noncovalent interactions. Hence, liquid droplets can act as reaction vessels, allowing for selective and sensitive detection of biomarkers through various bioanalytical techniques. Moreover, certain cargos preloaded in the droplets, such as receptors, enzymes, and DNA, can be utilized to promote preferential uptake and enrich molecular biomarkers of interest into the liquid droplets. Gong and co-workers elegantly developed liquid droplets composed of RNA-sensing DNA nanostructures joined via linker molecules, which capture specific tumor-derived miRNA.^4^ These DNA droplets exhibited three-phase separation behavior upon detecting miRNA of interest, which could subsequently be visualized using confocal fluorescence microscopy.

This strategy provides a versatile and adaptable platform for sensitive and specific biomarker detection, paving the way to early diagnosis of diseases without the need for invasive procedures and improving the patient’s prognosis. Further investigations are still needed to optimize and functionalize the droplets, achieve precise control over kinetics, enable high-throughput *in situ* detections, and many other aspects.

## Immunomodulation

The dynamic nature, spatial organization, and temporal control offered by liquid droplets make them an ideal platform for serving as artificial cells. Among them, artificial antigen-presenting cells (aAPCs) that promote robust *ex vivo* T-cell activation and expansion are tremendously attractive. The droplets can be engineered to spatially organize signaling molecules to create a localized T-cell activation and expansion microenvironment.

Given that the formation of T-cell receptor nanoclusters and the subsequent multivalent interactions are crucial for T-cell activation, we envision that supramolecular liquid droplets, formed through parallel alignment of elongated supramolecular polymers near thermodynamic equilibrium, could be potentially employed as an attractive aAPC.^5^ These droplets can be functionalized with various T-cell activating antibodies and cytokines through a modular approach. The fluidity shall provide a large surface area of cell contact, high loading capacity, and dynamic spatial rearrangement of T-cell activating cues, facilitating multivalent interactions and ultimately promoting robust T-cell activation and downstream signaling. However, the potential and applicability of such droplets as novel aAPCs for immunotherapy have yet to be elucidated.

## Metastable Liquid–Solid Transition as Neurodegenerative Disease Models

The metastable transition of liquid condensates into solid-like states is responsible for devastating neurodegenerative diseases, such as amyotrophic lateral sclerosis and frontotemporal dementia. Model systems that undergo liquid–solid metastable transition would help to map the energy landscape and unravel the underlying mechanisms behind aging, thereby deepening our understanding of the pathophysiology of various neurodegenerative diseases.^6^

Multicomponent supramolecular droplets comprising a diverse set of weak, transient interactions hold great promise to mimic both the structural and dynamic properties of biomolecular condensates. We recently reported a dilution-induced gel–sol–gel–sol cascade transition using supramolecular polymers and surfactants through competitive pathways. The *in situ* formation of supramolecular transient droplets was observed upon diluting on a functional supported lipid bilayer (SLB).^7^ The concentration gradient further promotes supramolecular polymerization from the interface of the metastable droplets, and over time the resulting fibers undergo coalescence to fabricate condensed fibril droplets. The unique system provides a detailed model for the physicochemical properties of different states (liquid, gel, and solid-like) in two distinct concentration regimes. Therefore, supramolecular droplets are potential model systems for systematic investigations of phase separation and phase transition in natural systems.

## Drug Discovery for Condensate-Modifying Therapeutics

Rapidly growing discoveries show that condensates’ aberrant locations, compositions, or physical properties are associated with various human pathologies, including cancer and degenerative disorders. Several encouraging reports emerged which show that (1) a set of small antineoplastic drugs is selectively concentrated in specific protein condensates that are driven by physicochemical properties independent of the drug target,^8^ (2) the physicochemical properties or phase behaviors of condensates can be altered by small molecule drugs to treat currently undruggable diseases,^9^ although the exact mechanisms of actions are not fully elucidated. Hence, a fundamental understanding of the interactions between drugs and the physicochemical environment of diverse biomolecular condensates, currently lacking for most drugs, may provide opportunities for previously unexplored drug discovery approaches.^10^

However, due to the complexity and technical limitation, systematic intracellular investigation of each interaction with different biomolecular condensates is difficult to identify. Thus, the *in vitro* reconstituted condensates from various scaffold proteins and/or nucleic acids serve as model systems for deciphering physicochemical codes implicated in the drug-condensate interactions. The Rosen group made great efforts in studying the partitioning of a library of 1700 small molecule metabolites and drugs into four condensates, using mass spectrometry with validation by fluorescence microscopy.^11^ It turned out that physical properties, rather than stereospecific interactions, drive the partitioning. The discovery is highly appreciated, yet the chemical grammar of drugs could be better addressed to arrive at the chemical rationales.

Besides the reconstituted condensate, synthetic liquid droplet models capturing some critical elements of the biomolecular condensates, such as heterogeneous inner structures, will aid in understanding the impacts of physicochemical properties of heterogeneous phases toward internal dynamics and biological activities, as well as guide the development of innovative drugs for condensate-modifying therapies. An elegant example was given recently by the Ulijn group, where they engineered a three-component system consisting of an oligo-arginine, adenosine triphosphate, and an amphiphilic peptide with both amyloid domain and oligo-arginine, enabling the coexistence of the self-assembled fibers inside of liquid droplets, through precise control over the ratios of the three components.^12^ The supramolecular system composed of limited types of molecules, each with well-defined interaction elements, can help isolate key molecular parameters to unravel how multivalent physical interactions drive drug partitioning into the droplets, and explore the alteration of physical properties upon the drug partitioning. The knowledge arising from this convergence research will advance our understanding of biomolecular condensates and provide innovative design principles for formulating novel drugs for condensate-modifying therapeutics.

## Outlook

Liquid droplets have embarked on interdisciplinary research to understand, mimic, and apply. For its true application as biomaterial, insights into the “structure–dynamics–biological function” relationship of biomolecular condensates as well as biotechnological advancements to enable precise control and manipulation of liquid droplets in real-time, is essential.
